# A Petri Net and LSTM Hybrid Approach for Intrusion Detection Systems in Enterprise Networks

**DOI:** 10.3390/s24247924

**Published:** 2024-12-11

**Authors:** Gaetano Volpe, Marco Fiore, Annabella la Grasta, Francesca Albano, Sergio Stefanizzi, Marina Mongiello, Agostino Marcello Mangini

**Affiliations:** Department of Electrical and Information Engineering, Polytechnic University of Bari, 70126 Bari, Italy; gaetano.volpe@poliba.it (G.V.); marco.fiore@poliba.it (M.F.); annabella.lagrasta@poliba.it (A.l.G.); f.albano5@studenti.poliba.it (F.A.); s.stefanizzi2@studenti.poliba.it (S.S.); agostinomarcello.mangini@poliba.it (A.M.M.)

**Keywords:** intrusion detection systems, neural network model, Petri nets

## Abstract

Intrusion Detection Systems (IDSs) are a crucial component of modern corporate firewalls. The ability of IDS to identify malicious traffic is a powerful tool to prevent potential attacks and keep a corporate network secure. In this context, Machine Learning (ML)-based methods have proven to be very effective for attack identification. However, traditional approaches are not always applicable in a real-time environment as they do not integrate concrete traffic management after a malicious packet pattern has been identified. In this paper, a novel combined approach to both identify and discard potential malicious traffic in a real-time fashion is proposed. In detail, a Long Short-Term Memory (LSTM) supervised artificial neural network model is provided in which consecutive packet groups are considered as they flow through the corporate network. Moreover, the whole IDS architecture is modeled by a Petri Net (PN) that either blocks or allows packet flow throughout the network based on the LSTM model output. The novel hybrid approach combining LSTM with Petri Nets achieves a 99.71% detection accuracy—a notable improvement over traditional LSTM-only methods, which averaged around 97%. The LSTM–Petri Net approach is an innovative solution combining machine learning with formal network modeling for enhanced threat detection, offering improved accuracy and real-time adaptability to meet the rapid security needs of virtual environments and CPS. Moreover, the approach emphasizes the innovative role of the Intrusion Detection System (IDS) and Intrusion Prevention System (IPS) as a form of “virtual sensing technology” applied to advanced network security. An extensive case study with promising results is provided by training the model with the popular IDS 2018 dataset.

## 1. Introduction

Cybersecurity is one of the most demanding challenges that companies have faced over the last few years. According to a recent survey, not only big companies are continuously under threat, but also small companies (SMBs), which are even more vulnerable due to the limited available resources and the lower complexity of their defense infrastructures [1]. The main goal of a cybersecurity framework is to protect an enterprise network and its devices from unauthorized access or criminal use to ensure the confidentiality, integrity, and availability of information.

Cybersecurity techniques include anti-virus software, firewalls, and Intrusion Detection Systems (IDSs) that protect networks from internal and external attacks [2]. In particular, an IDS is a device or a software that continuously monitors inbound and outbound network traffic for abnormal activities or policy violations [3]. A detected anomaly is normally reported to an administrator, and forensic evidence is collected for an immediate or posthumous analysis. However, most of the standard approaches that are based on statistical methods suffer from a high false alarm rate that force administrators to set higher detection thresholds so as not to be burdened by too many notifications. As a result, some potentially harmful attacks have a high probability of being ignored.

In the recent literature [4], IDSs have adopted new methods based on Machine Learning (ML) techniques to overcome the limits of legacy approaches. These methods make use of existing labeled datasets made of raw network traffic data collected over time. Indeed, ML-based IDSs have at least two main advantages over standard techniques: (i) they do not rely on domain knowledge; therefore, they are easy to design and build; (ii) when fed with a proper training dataset, balanced and adequate in size, they provide the necessary generalization level to detect both known and unknown attacks. More specifically, when big data are concerned, a deep learning approach is more suitable. Deep learning is a branch of ML characterized by its dense structure made of multiple hidden layers [5]. Compared with traditional ML techniques, deep learning can automatically learn feature representation from raw data and then output results.

Cybersecurity challenges grow more sophisticated; leveraging advanced machine learning models within distributed systems has shown promise in threat detection and mitigation. In recent studies, [6] explored a robust, ensemble machine learning model aimed at mitigating cyberattacks within cloud–fog architectures, demonstrating how distributed model frameworks enhance the resiliency of threat detection mechanisms in dynamic environments.

However, when it comes to network traffic analysis, the time correlation of sequential packets is not negligible, and, therefore, it must be considered. One simple example is Distributed Denial of Service (DDoS) attacks [7]. DDoS attacks are a subclass of Denial of Service (DoS) attacks. A DDoS attack involves multiple connected online devices, collectively known as a botnet, which are used to overwhelm a target website with fake traffic [8].

Several research approaches to manage DDoS attacks based on ML solutions have been proposed in the literature: [9] introduced a user behavior analytics (UBA)-based solution utilizing LSTM neural networks to address DDoS threats, underscoring the effectiveness of sequence-based models in recognizing and reacting to anomalies in real-time.

In respect to those kinds of attacks, Long Short-Term Memory (LSTM) networks, a special class of Recurrent Neural Networks (RNN), have been investigated in the literature [10,11]. RNNs are networks designed for sequential data and are widely used in natural language processing (NLP) and time series analysis [12]. LSTMs are a subclass of RNNs in which the problem of balancing outdated and recent data is solved by combining short-term and long-term memory to generate the current memory state [13].

However, in this context, the analysis of simple sequential network packets has the main disadvantage in the complexity of determining the right batch size, and it does not often performs as expected in detecting intrusions. As a result, a higher-level solution should be investigated. Cybersecurity challenges are growing more sophisticated, and leveraging advanced machine learning models within distributed systems has shown promise in threat detection and mitigation. In recent studies, Nocera et al. (2022) [6] explored a robust, ensemble machine learning model aimed at mitigating cyberattacks within cloud–fog architectures, demonstrating how distributed model frameworks enhance the resiliency of threat detection mechanisms in dynamic environments.

Despite the effectiveness of traditional LSTM-based IDSs in detecting network anomalies, several limitations remain unaddressed. Current LSTM models are primarily focused on anomaly detection without offering robust mechanisms for managing real-time network traffic, often leading to high false-positive rates in complex environments. Additionally, Petri Nets have been used for modeling decision-making processes, but their applications have been largely limited to simpler rule-based scenarios, lacking the integration with advanced ML models necessary for handling the sequential nature of network data. These gaps underscore the need for a more comprehensive approach that combines the predictive power of LSTM with the structured flow control of Petri Nets, enabling both accurate detection and real-time mitigation of threats. This dual-layered methodology aims to reduce false positives and enhance the system’s responsiveness to evolving network conditions.

In this paper, a high-level IDS system is proposed, in which the dataset is made of labeled groups of sequential packets. The instances are sequential in time and include cumulative data such as the minimum size of packets in forward direction, the total size of packets in backward direction, and the mean and standard deviation time in which a flow was active before becoming idle. Moreover, each record is labeled in the last field as malicious or not, in a binary fashion. An LSTM network is trained on the dataset by adjusting the hyper-parameters to enable the model to recognize attacks based on the analysis of network flow. In addition, the real-time detection is combined with an Intrusion Prevention System (IPS) to prevent the malicious traffic flowing through the enterprise network. An IPS is a security tool that continuously monitors a network for malicious activity and takes actions, such as blocking or dropping packets, to prevent it [14]. In the proposed network, the IPS is modeled using a Petri Net (PN) where the places represent packet buffers at various levels, and the transitions control packet flow through the network based on the IDS output. In contrast to traditional LSTM-based IDSs, which focus primarily on detection, the integration with Petri Nets allows for real-time control of network packet flow. This approach not only detects but also immediately mitigates identified threats, which is a significant advancement over standalone LSTM models. The Petri Net framework provides a clear and formalized way to manage packet flow decisions, improving the system’s responsiveness to network anomalies. An extensive case study is provided by using a popular IDS dataset [15] to demonstrate the effectiveness of the proposed framework. In detail, simulation results show that all malicious attempts are effectively blocked, whereas a residual false positive rate of about 18% of erroneously blocked benign packets is observed.

The system described is particularly suitable for applications in virtual environments or distributed Cyber–Physical Systems (CPS), where security is critical. The combination of LSTM and Petri Nets offers real-time threat detection, safeguarding user interactions and sensitive data. In environments such as virtual reality (VR) or augmented reality (AR), where sensitive data are frequently exchanged, an effective IDS-IPS system can act as a preventive security measure to ensure the protection of user information and interactions. The proposed architecture, based on LSTM and Petri Nets, can be viewed as a virtual sensing system capable of “perceiving” and responding in real time to anomalous behaviors within the network. This approach is akin to the role that physical sensors play in virtual and augmented reality environments, where real-time data acquisition and processing are essential.

The experimental results demonstrate that our LSTM–Petri Net hybrid approach significantly improves upon these baselines. Specifically, our framework achieves a detection accuracy of 99.71% compared with an average of 97% for traditional LSTM-only models.

Summarizing, the main contributions of the proposed paper are as follows:

- A ML-based approach to identify and discard malicious traffic in a real-time fashion is proposed. The proposed algorithm encompasses an LSTM network that acts as an IDS and is able to catch the time correlation of the network packets flowing through the corporate network and identify incoming dangerous packet sequences;
- A PN to check the packet flow throughout the network and choose whether to accept or block a packet is modeled. Firstly, the PN acts as a buffer by storing a predefined bunch of packets. Then, it predicts the next packet, acting as an IPS, and decides to discard or let through the whole buffer according to the output of the LSTM-based IDS;
- The combination of the two strategies constitutes a high-performance IDS-IPS framework that is able to identify and block malicious packet sequences in real-time and drastically lower the risk of compromising the corporate network. This combination is not extensively explored in the current literature.

The rest of this paper is organized as follows: Section 2 provides the background in intrusion detection, intrusion prevention, and the related state-of-the-art in recent literature. Section 3 introduces the proposed framework and the details of the IDS LSTM model and the PN-based IPS. Section 5 describes the case study, and Section 6 shows the simulation results. Finally, Section 7 draws conclusions and outlines future work.

## 2. Related Work

The idea of IDSs was first presented in 1980 by James Anderson [16]. Then, because of improvements to the network and the processing capacity of its components, and thanks to the development of ML techniques, IDS is now an integral component of the security of many systems. Most ML algorithms were examined throughout IDS development. Deep learning algorithms offer additional options to address the problem of false alarm rates and the lack of high accuracy.

### 2.1. Deep Learning Methods in Intrusion Detection

In contrast to traditional ML, the latest approach referred to as deep learning has shown state-of-the-art performance on many problems [17,18] including intrusion detection. Deep learning provides automated tools for deep feature extraction. It gives a better representation of data for generating improved models.

Park et al. propose a graph-based intrusion detection and classification system, named G-IDCS, which aims to enhance the security of the in-vehicle controller area network (CAN) protocol, reducing the number of CAN messages required for detection by more than 1/30 and improve the accuracy of combined attack detection by over 9% compared with an existing intrusion detection method that uses graph theory [19].

A deep learning-based novel method to detect cybersecurity vulnerabilities and breaches in cyber–physical systems is proposed in [20]. In contrast with the unsupervised and deep learning-based discriminative approaches, the authors propose a generative network to detect cyber threats in IoT-driven Internet Industrial Control Systems (IICs) networks.

A deep learning approach can be useful in the context of account compromisation, caused by the ability of the spammers to spread malicious messages by exploiting the trust relationship established between account owners and their friends. In this sense, an optimized nonsymmetric deep autoencoder for unsupervised feature learning, which reduces the required human interaction levels in the selection and extraction of features, is presented in [21]. Similarly, to minimize the amount of human expert involvement needed during the feature selection process, Boahen et al. propose a combination of an unsupervised deep learning technique with a heuristic way of class separation, proceeded by an upgraded convolutional neural network for feature selection for classification [22].

A Hybrid Strategy Improved Sparrow Search Algorithm (HSISSA) is discussed in [23], specifically for feature selection and model optimization in Intrusion Detection Systems. This method employs a hybrid circle–piecewise map for population initialization to enhance the uniformity of the initial distribution and incorporates the spiral search method from the vulture search algorithm and Levy’s flight formula to update the positions of discoverers and scouters, thereby expanding the search range.

A novel IDS has been developed and implemented using the Maximum Dependence Maximum Significance algorithm for selecting the minimal number of attributes of Knowledge Discovery and Data (KDD) dataset and a new K-Nearest Neighbors-based algorithm is proposed for classifying the dataset [24].

### 2.2. Recurrent Neural Networks (RNNs) in Intrusion Detection

RNN has become one of the most widely used approaches in deep learning for carrying out classifications and other evaluations on data sequences, building on today’s research in the domain of intrusion detection [25,26]. Due to the recurrent fashion, these neural networks are connected and better suited for processing sequential data. Unlike conventional feed-forward networks, RNNs can remember what they have learned in the past and base their decisions on this information. However, RNNs also have drawbacks that prevent them from learning long-term dependencies. Hochreiter and Schmidhuber came up with the concept of the Long Short-Term Memory (LSTM) to solve this issue [27]. A model for the detection of network intrusions using LSTM–RNN was presented by Kim et al. [28]. Their model was trained using the dataset from the KDD Cup 1999, and results provided high accuracy, showing the usefulness of DL for IDS. Using LSTM and RNN, Fu et al. suggested a system for the intelligent detection of network attacks [29]. The architecture of the system is made up of three layers: the input layer, the mean pooling layer, and the regression layer. When trained with the NSL-KDD dataset, the methodology gave satisfactory results in terms of performance. In [30], Staudemeyer introduced an LSTM model for the detection of network intrusions. When modelling network traffic as a time series, the author considered both typical user behavior and that of malevolent users. To assess the efficacy of the strategy, the model was educated using the DARPA and KDD Cup 1999 datasets, and experiments were conducted using various network topologies. The research also looked at several feature sets to identify assaults and develop training on networks that were tailored to certain attack types. Le et al. [31] developed an IDS classifier by using a recurrent neural network strategy. The authors conducted research on a total of six distinct optimizers for LSTM–RNN. The authors believe that out of the six optimizers, Nadam is the one that is most appropriate. In comparison to other works, the performance it achieved in detecting intruders was rather satisfactory. In their study, Yakubu Imrana and colleagues present a bidirectional Long Short-Term Memory (BiDLSTM)-based IDS [32]. They use the NSL-KDD dataset to train and test the performance of the model. This study displays a decrease in false alarm rate in comparison to the current models and provides a greater detection accuracy for U2R and R2L attacks in comparison to the standard LSTM.

### 2.3. Petri Nets (PNs) in Intrusion Detection

In the field of information security, several studies on modeling and assessing the performance of systems using PNs have been undertaken by researchers. Mixia et al. provided the system architecture of the network security scenario and modeled it using the Colored Petri Nets (CPN) to examine the network security from the perspective of the system [33]. Hicham et al. came up with an innovative method to identify network security conflicts in a more generalized manner, and they described it using CPN so that automated conflict analysis could be carried out [34]. Fuzzy Petri Nets (FPN) were the method that Hwang et al. [35] used to explain the inference principles of computer forensics. They retrieved and examined the data that had been obtained from the hacked systems to extrapolate information on the breach. A formal reference behavior model was given by Voron et al. [36] using PNs. This model may automatically create host-based IDS from software sources. A new IDS architecture that is based on partly ordered events and a unique detection algorithm that matches the intrusion signature with PNs was developed by Balaz et al. [37].

### 2.4. Graph Learning and Federated Learning in Intrusion Detection

Graph learning methods have gained attention in intrusion detection due to their ability to model complex relationships between network entities. Federated learning, on the other hand, provides a solution to address data privacy concerns in distributed systems while enabling collaborative model training.

Jianping et al. [38] propose an attention-based graph neural network (GNN) designed for detecting cross-level and cross-department network attacks. By constructing graph structures based on chronological network traffic logs and introducing a Federated Graph Attention Network (FedGAT) model, this approach enhances the detection accuracy while maintaining data privacy. This is particularly relevant in distributed environments, such as IoT networks, where centralized data collection poses privacy risks.

Similarly, Tran et al. introduce a novel preprocessing technique for network flow data and a graph model based on graph neural network (GNN) and SAGEConv [39]. This approach addresses the limitations of standardizing input data for Intrusion Detection Systems and demonstrates significant improvements in the detection of malicious network flows. By initializing graph nodes with flow features and defining edges through IP relationships, this method provides a scalable and effective solution for real-world applications.

In stark contrast to existing research in the field of IDSs, this work introduces an innovative and encompassing methodology that distinguishes itself in several key aspects. While traditional approaches, as seen in the study of Anderson et al. [16], often suffer from a lack of clear temporal correlation, the proposed solution not only leverages the power of LSTM supervised artificial neural network models but also integrates the entire IDS architecture into a PN framework. This dual-layered approach is a departure from the conventional separation of ML and PN modeling, presenting a holistic and synergistic solution for real-time intrusion detection in corporate networks and distinguishing itself in a concrete way from prior research, as underlined, for example, in Le et al.’s work [31], who merely explored LSTM applications in intrusion detection. Moreover, the proposed model explicitly addresses the temporal correlation in training sets, a critical aspect often overlooked in the literature. By considering consecutive packet groups as they flow through the network, this approach enhances the adaptability of the IDS to the continuous nature of network traffic. The distinctiveness of this work extends to its pragmatic implementation and empirical validation. In contradistinction to certain related works that lack concrete application or validation, this paper conducts an extensive case study employing the widely recognized IDS 2018 dataset. This empirical evaluation not only demonstrates the efficacy of the approach but also provides a tangible and relevant benchmark for comparison. Additionally, this paper critically compares the proposed approach with traditional ML techniques, shedding light on the advantages of the proposed deep learning-based model over traditional methods in terms of accuracy and false alarm rates. In summary, this work represents a significant advancement in this field, offering a unique and effective solution to the challenges faced by traditional IDS methods and presenting a comprehensive analysis of related works for context and comparison.

Table 1 shows different literature works based on the IDS 2018 dataset and their main contribution in the topic of IDSs.

## 3. Materials and Methods

**Figure 1:**
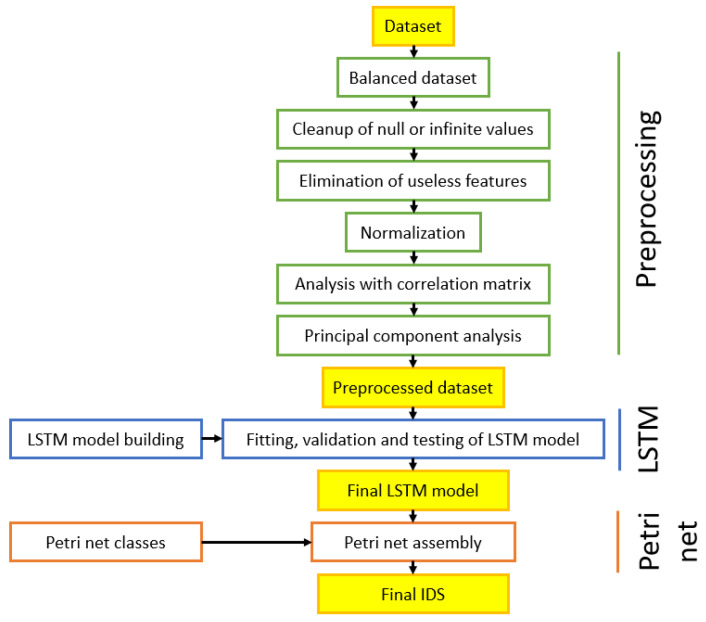
The overview of the proposed method.

This section shows the main technologies and approaches used in this paper, analyzing the potentiality of each component. Section 4 shows the actual implementation of the described tools. The flow chart of the LSTM-based IDS proposed in this work is shown in Figure 1 and described with Algorithm 1.

The algorithm is designed to combine the strengths of sequential data analysis through LSTM and real-time packet decision-making with Petri Nets. A detailed analysis of the algorithm’s components is presented below:

- First, the data are preprocessed by loading the dataset, balancing benign and attack packets, handling missing or infinite values, and standardizing features. Highly correlated features are removed, and Principal Component Analysis (PCA) is applied to reduce dimensionality.
- The preprocessed data are then prepared for LSTM training by converting them into sequences of window size 50 and splitting them into training, validation, and test sets.
- An LSTM model is defined, compiled with binary cross-entropy loss and the Adam optimizer, and trained using the training and validation data. The trained model is evaluated on the test set to assess its accuracy and precision.
- A Petri Net is defined with places representing packet states and transitions controlling packet flow based on LSTM predictions. The network structure includes connections between places and transitions to model decision-making.
- The Petri Net executes by simulating packet arrival and utilizing LSTM predictions to decide whether packets are benign or malicious. The packets are either stored in a non-blocked list (if benign) or a blocked list (if malicious), and the process is repeated for multiple sets of data.

**Algorithm 1** LSTM and Petri Net Workflow for IDS  1:**Data Preprocessing:**  2:Load dataset  3:Balance benign and attack packets  4:Labels mapping (0 for benign, 1 for attack)  5:Handle missing and infinite values  6:Standardize features  7:Remove highly correlated features  8:Apply PCA to reduce dimensionality  9:**Prepare Data for LSTM:**10:Convert data to sequences of window size 5011:Split data into training, validation, and test sets12:**LSTM Model Training:**13:Define LSTM model with layers14:Compile model with binary cross-entropy loss and Adam optimizer15:Train model with training and validation data16:Save trained model17:**LSTM Model Evaluation:**18:Evaluate model on test data19:Calculate confusion matrix and classification report20:**Petri Net Definition:**21:Define places: p1,p2,p3,b,a,n22:Define transitions: t1,t2,t3,t423:Define arcs:24:t1: b→p125:t2: p1→p2 & p1→p326:t3: p2→a & p2→b27:t4: p3→n & p3→b28:**Petri Net Execution:**29:Initialize Petri Net with test data30:**for** i=1 to 10 **do**31:   Fire transition t1 to simulate packet arrival32:   **for** j=1 to 50 **do**33:     Wait for next packet34:     Fire transition t1 to simulate packet arrival35:   **end for**36:   Fire transition t2 to predict packet flow using LSTM37:   **if** p2 has token and p3 does not **then**38:     Fire transition t4 (Benign packet flow)39:     Store packets in non-blocked list40:   **else**41:     Fire transition t3 (Attack packet flow)42:     Store packets in blocked list43:   **end if**44:   Move to next set of packets45:**end for**

The preprocessing phase consists of reading and balancing the dataset. The dataset is initially divided into two classes: records labelled as benign and malignant. To balance the dataset, the ratio between the number of malignant and benign samples is calculated. When this ratio is greater than 1, the samples in the malignant class are reduced to equal the number of benign samples, and vice versa. In our case, since the malignant packages were more numerous than the benign ones, a reduction of the former was performed. It is important to emphasize that in the subsequent phase of splitting into training and test sets, the samples are not randomly mixed, because it is essential to preserve the time dependency between the data for proper training of the LSTM model. Then, the dataset is optimized through a cleaning of null or infinite values, the elimination of useless features, and the normalization. Finally, a correlation matrix analysis and a Principal Component Analysis (PCA) are applied to obtain the preprocessed dataset. An LSTM model is built and then validated and tested with the preprocessed dataset. The final Intrusion Detecion System is modeled with a PN to analyze the information flow.

### 3.1. Balanced Dataset

The dataset to use for this work must contain information related to packets detected by a proprietary third-party IDS. This is essential because labeled data are needed to train the LSTM model for the classification between benign and malicious packages. The dataset must contain temporal dependencies between the information contained within it, since the LSTM model, for which this dataset is used, needs to know these dependencies between data; thus, the model can choose which information to keep and which to discard for the determination of the current state.

### 3.2. Data Analysis

The main goal is to perform a binary classification between malicious and benign packages. Typically, datasets used to build systems similar to the one shown in this paper contain several values for the target feature. Therefore, a binary mapping of the data is needed, replacing the target feature with the following:

- 0: if the target feature is “Benign”;
- 1: if the target feature is different from “Benign”.

Conversion with numeric values must be performed to make the data-frame compatible for PCA. The data-frame could also contain NaN or infinite values, so it is necessary to substitute those values. Null values can be replaced with “0”, while infinite ones with “1”.

### 3.3. Normalization

The normalization technique is used in this work as one of the data preprocessing steps to make data compatible with PCA. By using this technique, it is possible to identify a new range of data from an already existing range.The normalization technique applied in this work is the Z-score normalization, which provides normalized values from the original unstructured data, using concepts such as mean and standard deviation. The original data can be normalized by Equation (1):

(1) $$x_{i}^{\prime }=\frac{x_{i}-\bar{E}}{std(E)}$$

where the following is true:

$x_{i}$ is the value relating to row *E* and the *i*-th column;

$x_{i}^{\prime }$ is the Z-score normalized value relating to row *E* and the *i*-th column;

$\bar{E}=\frac{1}{n}\sum_{i=1}^{n}x_{i}$ is the average value of row *E*;

$std\left(E\right)=\sqrt{\frac{1}{\left(n-1\right)}\sum_{i=1}^{n}{\left(x_{i}-\bar{E}\right)}^{2}}$ is the standard deviation of row *E*.

### 3.4. Correlation Matrix

**Figure 2:**
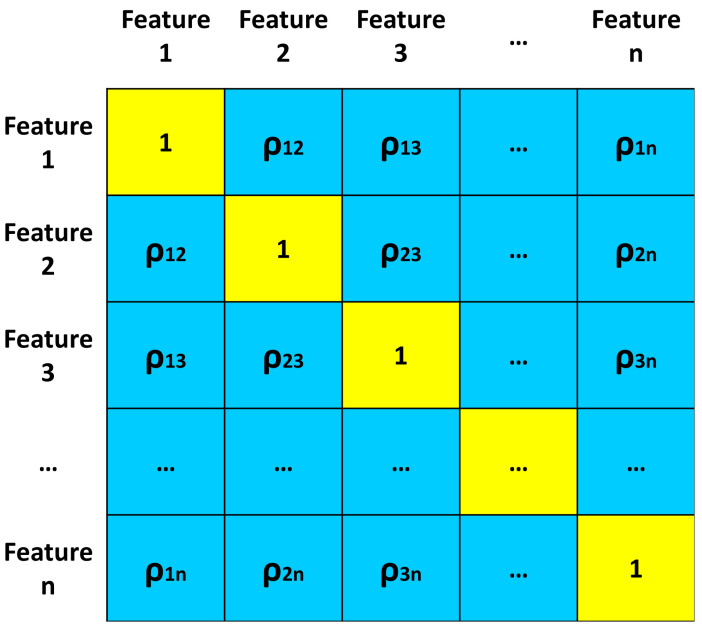
Structure of a correlation matrix.

An analysis with a correlation matrix is performed because of the presence of too many features in the data-frame. The matrix is calculated based on the data-frame made up of all the values of the training features (excluding the target feature). A correlation matrix is a symmetric matrix with unit diagonal. The elements (*i*, *j*) of the correlation matrix are called “correlation indices” or “correlation coefficients” and indicate the correlation between features *x*_*i*_ and *x*_*j*_. The structure of the correlation matrix is illustrated in Figure 2, where, ρ_*ij*_ are the correlation coefficients.

The matrix has 3 sections:

- Main diagonal: the elements are all equal to 1. For the illustrated work, these coefficients are not useful for the analysis and therefore are not considered;
- Upper right triangle: it contains the correlation coefficients of each feature with the others;
- Lower left triangle: the same and identical correlation coefficients of the upper right triangle are contained.

Therefore, since the correlation coefficients within a matrix appear twice, the correlation coefficients of only one triangle within the correlation matrix are useful in the analysis. The Pearson method was used as a correlation method [48]. Therefore, within the matrix, the correlation coefficients can assume values between −1 and 1.

### 3.5. Principal Component Analysis

**Figure 3:**
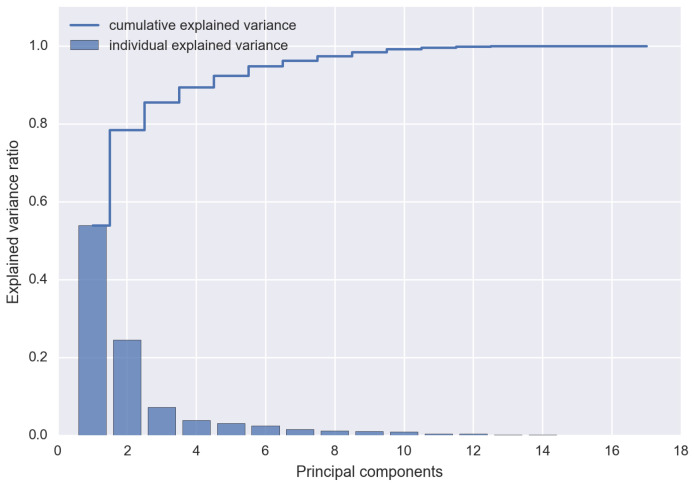
Example of Scree-plot of PCA. The explained cumulative variance of the starting components and the explained individual variance of each starting component are shown.

The PCA is a mathematical algorithm that allows one to reduce the dimensionality of data, allowing one to maintain the information load in the dataset [49]. It is used when, within the dataset, there are many related features, and it is needed to reduce their number, losing the least amount of information. For this reason, PCA is applied in this work. In fact, it allows one to carry out the dimensionality reduction by identifying the directions (principal components) along which the variation of the data is maximum. Therefore, the objective of the PCA is to maximize the variance, calculating the weight to be attributed to each starting feature, in order to be able to concentrate them in one or more new features (principal components), which will be a linear combination of the starting features. Figure 3 shows an example of scree-plot of PCA.

The number of principal components to extract is a crucial step of PCA. It depends on how many features have been included in the PCA and how similar they are to each other. In fact, the more they are related, the lower the number of principal components necessary to obtain a good knowledge of the starting features. Conversely, the less they are correlated, the greater the number of principal components to be extracted in order to have accurate information about the dataset.

The method used in this work for the extraction of the number of principal components is the Scree-plot [50]. This method is based on a graph in which the values of the eigenvalues are shown on the vertical axis, and on the horizontal axis there are all the possible components to be extracted (which will be as many as the starting features). By joining the points, a broken line is obtained, which in some parts will have a concave shape and in others a convex one. Following this criterion, the number of principal components to be extracted is the one that coincides with the change in slope, i.e., with the elbow of the curve (about 90%), after which the broken line generally tends to flatten.

Table 2 shows the data structure before the application of PCA, while Table 3 shows the data structure before PCA.

### 3.6. LSTM

A neural network is used to build the model that can distinguish between benign and malicious packets. The Deep Learning model used is that of LSTM, which belongs to the class of recurrent neural networks (RNNs).

RNNs are models that store previous states and use them to compute the current state [51]. For this, the performance of RNNs with sequential data are high, and they are perfect to use when it is necessary to consider the context of the data in the training phase. To ensure this, the RNN layers are not independent of each other. In fact, RNNs are characterized by a cyclic connection, which allow to update the current state based on the current input data and previous states [52]. However, they are unable to connect information if the gap between the input data is large. This problem is known as “long-term dependencies”. In order to address it, Sepp Hochreiter and Jürgen Schmidhu-ber [27] proposed LSTM, in particular LSTM cells.

**Figure 4:**
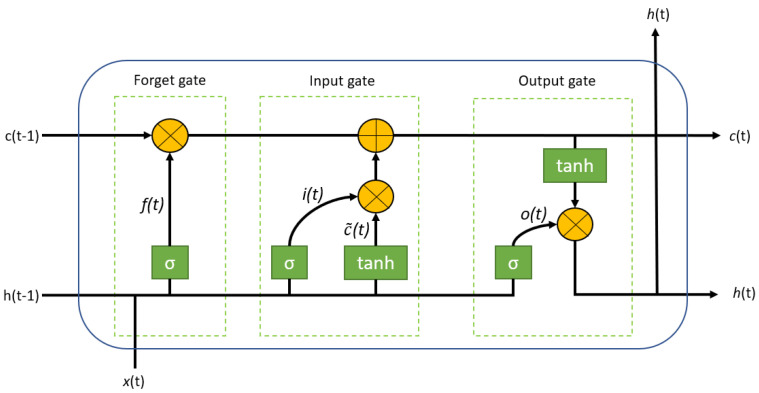
Structure of an LSTM cell with forget gate.

The LSTM cells introduce the “gates” that enable the presence of “residual memory”. The structure that these cells assume is illustrated in Figure 4, where σ is a logic function, tanh is a hyperbolic tangent function, ⊕ represents the operation of addition between elements, and ⊗ represents the operation of multiplication between elements.

The formulas that can be deduced from the connection described in Figure 4 are the following [53]:

(2) $$f_{t}=\sigma \left(W_{f}\cdot \left[h_{t-1},x_{t}\right]+b_{f}\right)$$

(3) $$i_{t}=\sigma \left(W_{i}\cdot \left[h_{t-1},x_{t}\right]+b_{i}\right)$$

(4) $${\tilde{c}}_{t}=tanh\left(W_{c}\cdot \left[h_{t-1},x_{t}\right]+b_{c}\right)$$

(5) $$c_{t}=f_{t}*c_{t-1}+i_{t}*c_{t}$$

(6) $$\sigma_{t}=\sigma \left(W_{o}\cdot \left[h_{t-1},x_{t}\right]+b_{o}\right)$$

(7) $$h_{t}=o_{t}*tanh\left(c_{t}\right)$$

So, the gates are 3:

1. Forget gate → through the sigmoid, it is decided which information will be discarded from the cell state. If the value of *f*_t_ is equal to 1, then the information is kept, while if the value of *f*_t_ is equal to 0, the information is discarded.
2. Input gate → determines what information will be stored in the cell state. The sigmoid determines which information *i*_t_ to update. The tanh creates a vector of $\tilde{c}$_t_ values. Then, multiplying *i*_t_ and $\tilde{c}$_t_, the new cell state *c*_t_ is received.
3. Output gate → the sigmoid determines the parts of the cell state to be brought to output (*o*_t_). The tanh processes the cell state *c*_t_. Then, multiply the two values obtained.

In this work, the resulting LSTM network is built using the keras sequential model [54], alternating LSTM-type layers with Dropout-type layers to prevent model overfitting. The input layer of the LSTM network accepts 50 packets; the penultimate layer has an output dimensionality equal to 1, since the classification treated in this paper is binary, while the last layer is of sigmoid type activation. In Table 4 the parameters of the LSTM are summarized.

The network consists of six hidden layers, each with 80 LSTM units, designed to capture complex temporal dependencies. To prevent overfitting, six dropout layers with a 30% deactivation probability are used, improving generalization. The six hidden layers increase the model’s learning capacity but require longer training times and are more prone to overfitting if not regularized properly. The training uses a batch size of 512, allowing for efficient processing of large data while managing memory requirements. The model trains over five epochs, balancing learning complexity and the risk of overfitting.

### 3.7. Petri Net

In this work, a PN is implemented to model the whole IDS. Indeed, PNs constitute a simple and powerful method to describe and analyze the flow of information (which, in the case of this work, are packets) and controls in systems [55].

A PN is described by the four-tuple:

(8) PN=(P,T,Pre,Post)

where *P* is the set of the places (circles), *T* is the set of the transitions (bars), Pre:P×T→N is the pre-incidence matrix and specifies arcs from places to transitions, Post:P×T→N is the post-incidence matrix and specifies arcs from transitions to places.

The dynamics of MAPNs is governed by two main rules, namely the “enabling rule” and the “state transition rule”. Before defining these rules, it is necessary to introduce the following concepts:

- Token: a place that contains tokens indicates that the condition corresponding to the place is met;
- Marking: a function M:P→N that associates a non-negative number to each place. *M* is also defined by a vector containing the number of tokens for each place. In other words, it is the distribution of tokens in places in the system and corresponds to the state of the PN;
- Initial marking $M_{0}$: the initial marking present at the beginning of the observation of the system.

Thus, the system $<PN,M_{0}>$ is described by the following rules, which govern its dynamics:

- Enabling rule: when an input place to a transition has the required number of tokens, the transition is enabled.
- State transition rule: only when a transition is enabled, it can fire and one or more tokens are removed from each input place (as many as the weight of its input edges) and one or more tokens are placed in each output place (as many as the weight of its output edges).

More in detail, the two cited rules can be formulated mathematically as follows:

- The enabling condition is met if
  (9) $$M>=Pre(t_{i})$$
- The state condition leads to the creation of a new marking $M_{i}$ after the occurence of transition $t_{i}$ at the marking $M_{i-1}$:
  (10) $$M_{i}=M_{i-1}-Pre\left(t_{i}\right)+Post\left(t_{i}\right)$$

## 4. Implementation

The proposed method consists of three main steps:

1. DATA PREPROCESSING: the “IDS 2018 Intrusion CSVs” datasets are imported, related to 16 February 2018 [15]. These data are preprocessed using different methodologies, including normalization, correlation matrix, and Principal Component Analysis;
2. LSTM MODEL: the LSTM model is created to allow the binary classification of data into “malign” and “benign”. Model fit is performed using data from the training set. Then, the model is validated using the data from the valid set. Subsequently, the model is evaluated on the test dataset, with a prediction phase.
3. PN: the IDS is made functional by building a PN. The main classes that compose the net and the methods to allow its operation are defined. Next, the network is assembled and run, showing the performance of the IDS.

### 4.1. Data Analysis

The set of all datasets in the “IDS 2018 Intrusion CSVs” is considered excessive for the model training. Therefore, only one of the datasets in this collection is used in this work. To allow for optimal performance of the LSTM model, it is necessary to balance the dataset, equating the number of benign rows to the number of malicious rows. A model trained with balanced data performs much better than a model trained with unbalanced data. Some features may not be useful for the purposes of this work; thus, they are removed.

To perform Z-score normalization, the features *x*_*i*_ and *x*_*j*_ whose correlation index ρ_*ij*_ is in absolute value greater than 0.8 are considered highly correlated:

(11) $$\left|\rho_{ij}\right|>0.8$$

The features *x*_*i*_ and *x*_*j*_ that satisfy this condition are eliminated from the dataframes containing the training and test values for all the features (except the target feature).

### 4.2. Petri Net

**Figure 5:**
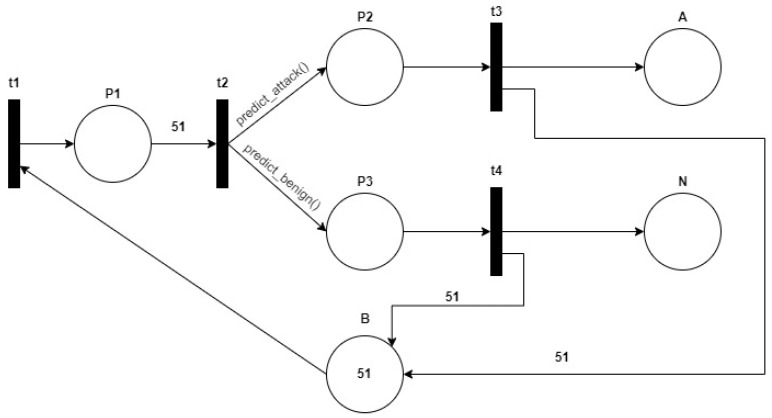
Structure of PN used in this work.

The PN used in this work, to assemble the entire IDS and to run it entirely, is the one shown in Figure 5:

The place B represents the buffer, i.e., the capacity of the entire network. It indicates that the total number of packets that must transit within the PN must be <=51. Therefore, it is used to be able to analyze a flow of 51 packets at a time; otherwise, packets would enter the network indefinitely, and it would not be possible to block the flows in real time. The place A represents a sort of “trash”, in which the packets that have been discarded and that are malicious are placed. In place N, however, there go all the packets that have not been discarded and which, therefore, are benign. The arc function “predict_attack()” is inserted on the arc leaving the transition t2 and entering it in place P2. This function, after having received 50 total packets, makes the prediction on the 51st. If the packet is classified as an attack, then one token is placed in P2. Conversely, if the packet is classified as benign, then no token is inserted into P2. The arc function “predict_benign()” is inserted on the arc leaving the transition t2 and entering it in place P3. This function, after having received 50 total packets, makes the prediction on the 51st. If the packet is classified as benign, then one token is placed in P3. Conversely, if the packet is classified as an attack, then no token is entered in P3. At this point, transitions t3 or t4 can be triggered. In particular, if one token is present in P2 (therefore no token is present in P3), then transition t3 is activated, and all 51 packets go to place A, so they have been discarded. Conversely, if one token is present in P3 (therefore no token is present in P2), then transition t4 is activated and all 51 packets go to place N, so they have not been discarded.

For the construction of the network, the following classes are defined:

- Place: for the definition of the characteristics of a place in the PN. It is characterized by name and number of tokens;
- Transition: for the definition of the characteristics of a PN transition. It is characterized by a name, a set of input arcs, and a set of output arcs;
- Net: for the definition of the characteristics of the PN. It is characterized by name, current marking, a set of transitions, and global_var. The global_var attribute is composed as follows:
  − global_var[0]: contains the entire preprocessed data-frame;
  − global_var[1]: contains the index (randomly extracted) of the first packet of the 50 packet flow to be considered for the prediction of the 51st;
  − global_var[2]: contains the “df_bloc” data-frame in which all blocked packets are kept track of;
  − global_var[3]: contains the “df_not_bloc” data-frame where all unblocked packets are tracked

As methods of this class, several have been defined:

− add_place(self, pl): with this method, a place is added within the network with its number of tokens;
− add_transition(self, trans): with this method, a transition is added inside the network, with the input and output arcs;
− add_input(self, trans, pl, weight = 1): with this method, an arc and its weight are inserted at the entrance to a transition and at the exit from a place;
− add_output(self, trans, pl, weight = 1): with this method, an arc and its weight are inserted out of a transition and into a place;
− fire(self, trans): with this method, the transition rule and enabling rule are implemented. In particular, for the transition t2, which has the predict_attack() and predict_benign() functions as output arc weights, the respective predict functions are executed.

It is necessary to define the function that allows the functioning of the network itself, that is, the start_net() function. For a total of 10 flows composed of 51 packets (of which the 51st is predicted), the entire PN is executed. First, transition t1 is fired, which allows the 51 packets to enter the network. Then, transition t2 is fired. Based on the number of tokens in P2 and P3, transition t3 or t4 is enabled. If the number of tokens in P2 is 1 (and therefore P3 has no token), then transition t3 is enabled and the 51 packets are inserted in place A; otherwise, if the number of tokens in P3 is 1 (and therefore P2 has no token), transition t4 is enabled and the 51 packets are inserted into place N. After repeating the process for 10 total streams of 51 packets at a time, various statistics are printed, including the following:

- Number of total blocked packets in place A;
- Number of total NOT blocked packets present in place N;
- Percentage of blocked attack packets;
- Percentage of blocked benign packets;
- Percentage of unblocked attack packets;
- Percentage of unblocked benign packages.

The PN is then built, using the previously defined classes:

- All places in the network are defined and added to it using the “add_place” method of the Net class.
- All transitions of the network are defined, which are added through the method of the Net class “add_transition”.
- All arcs of the network are defined. For each transition, the arcs entering it (with the “add_input” function) and the arcs leaving it (with the “add_output” function) are defined. In particular, the arc leaving transition t2 and entering P2 has the weight of the string predict_attack(net.global_var), whose function it represents is executed as soon as the fire() function is activated on this transition. In the same way, the arc leaving transition t2 and entering P3 has the weight of the string predict_benign(net.global_var), whose function it represents is executed as soon as the fire() function is activated on this transition.

## 5. Case Study

### Dataset Analysis

The system is implemented in Python using the Google Colab tool. The used version of the “CSE-CIC-IDS2018” dataset is optimized. The dataset comes from a collaborative project between the Communications Security Establishment (CSE) and the Canadian Institute for Cybersecurity (CIC). The main objective of this project is to develop a systematic approach to generate diverse and comprehensive reference datasets for intrusion detection based on the creation of user profiles that contain abstract representations of events and behaviors seen on the network. The profiles are combined to generate a diverse set of datasets, each with a unique set of characteristics, covering a part of the evaluation domain. The final dataset includes seven different attack scenarios: brute force, Heartbleed, Botnet, DoS, DDoS, web attacks, and insider network infiltration. The attacking infrastructure comprises 50 machines, and the victim organization has five departments with 420 machines and 30 servers. The dataset includes the captured network traffic and system logs of each machine, along with 80 features extracted from the captured traffic using CICFlowMeter-V3 (https://www.unb.ca/cic/datasets/ids-2018.html, accessed on 10 October 2024).

The main dataset, “CSE-CIC-IDS2018”, appears to have too large a number of rows to be able to use it optimally for the proposed IDS. For this reason, the use of its optimized version, i.e., “IDS 2018 INTRUSION CSVs”, is preferred. The available data are distributed in several CSV files, for a total of 6.89 GB. A total of 10 files is contained, each corresponding to a date on which the data contained in it were received/sent by the university servers. The CSV files are related to the following dates:

- 3 January 2018;
- 3 February 2018;
- 14 February 2018;
- 15 February 2018;
- 16 February 2018;
- 20 February 2018;
- 21 February 2018;
- 22 February 2018;
- 23 February 2018;
- 28 February 2018.

The “IDS 2018 Intrusion CSVs” datasets have 80 features, each corresponding to an entry in the IDS registration system that the University of New Brunswick has in place. Since their system classifies both received and sent traffic, there are columns for both cases.

The feature of interest for the purposes of this work, for the construction of the proposed IDS, is the “Label” feature. The possible values it can assume are different: the first value refers to not an attack; other values represent seven types of attack.

- Benign: the relative packet is not considered an attack, but it is judged as innocuous by the university’s IDS;
- Brute-force: tend to hack accounts with weak username and password combinations;
- Heartbleed: exploitation of a security bug for the theft of passwords and sensitive data;
- Botnet: a representation of a set of devices connected to each other, which, if infected with malware, can be used for the distribution of malicious programs;
- DoS: exhausting system resources by flooding it with illegitimate service requests;
- DDoS: Distributed Denial of Service. They are similar to DoS attacks but occur on a much larger scale as requests arrive from multiple sources at the same time;
- Web attacks: the website is scanned to find its vulnerabilities. Then, attacks are conducted on the site, such as SQL injection, command injection, and unlimited file upload;
- Infiltration of the network from inside: a malicious file is sent by e-mail to the victim and an application vulnerability is exploited. After the exploitation, the victim’s computer is used to scan the internal network and find other vulnerable computers.

In the preprocessing phase, all the rows that have a label value different from “Benign” are identified as an attack. This allows the creation of a binary classifier. The dataset is found to be unbalanced; therefore, balancing is carried out, equating the number of rows whose label feature is “Benign” to the number of rows whose label feature is different from “Benign”. Therefore, the number of rows in the overall data-frame, after balancing, is 893,544.

The label feature is of the object type and within the dataset used in this work (related to 16 February 2018); it assumes 2 unique values: “Benign” and “DoS attacks-Hulk”. The values assumed by this feature are replaced with the following:

- Zero if label is ”Benign;”
- One if label is different from “Benign.”

The substitution with numeric values is performed to make the data-frame compatible for PCA. The “Flow Pkts/s” and “Flow Byts/s” features can assume NaN or infinite values. NaN values are replaced with “0”, while infinite ones are replaced with “1”. Finally, some features are not useful for this work, so they are eliminated. They are as follows:

- Bwd PSH Flags;
- Fwd URG Flags;
- Bwd URG Flags;
- CWE Flag Count;
- Fwd Byts/b Avg;
- Fwd Pkts/b Avg;
- Fwd Blk Rate Avg;
- Bwd Byts/b Avg;
- Bwd Pkts/b Avg;
- Bwd Blk Rate Avg.

After the data analysis and cleaning phase, the data-frame is split into a training set (85% of the unsplit dataset) and a test set (15% of the unsplit dataset). In particular, the Numpy arrays X_train (containing all the values of the training set for all the features except Label), X_test (containing all the values of the test set for all the features except Label), y_train (containing all the values of the training set for the feature Label), and y_test (containing all test set values for the feature Label) are created. After that, X_train and X_test are normalized with Z-score normalization.

After normalization, a dimensionality reduction is run. First, the correlation matrix analysis is performed on X_train. The number of correlated features is equal to 50. Therefore, these features are eliminated from X_train and X_test. Therefore, features are reduced to 19, excluding the “Label” feature. The obtained features are described in Table 5.

**Figure 6:**
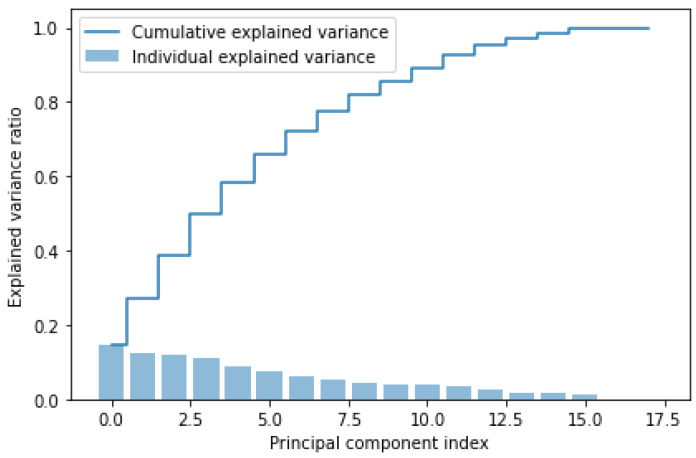
Scree-plot of PCA for the extraction of principal components. The explained cumulative variance of the starting components and the explained individual variance of each starting component are shown.

**Figure 7:**
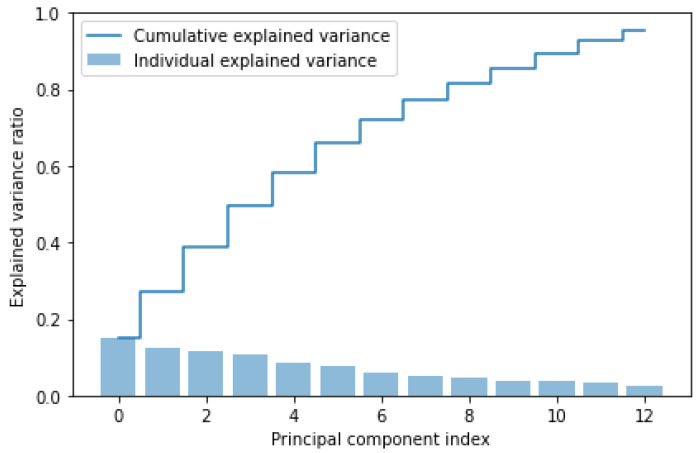
Scree-plot of PCA with 13 principal components. The explained cumulative variance of the starting components and the explained individual variance of each starting component are shown.

Subsequently, the dimensionality reduction is performed through Principal Component Analysis.The explained variance of each initial component and the cumulative sum of the variances are extracted to be plotted in the Scree-plot shown in Figure 6:

The elbow of the curve is located at 13 components, which correspond to the principal components to extract. The PCA with 13 principal components is rerun.The explained variance of each component and the cumulative sum of the variances are recalculated, and then they are plotted in the Scree-plot shown in Figure 7:

The objective of the PCA, i.e., maximizing the variance, is successfully achieved since the cumulative sum of the variances, after the PCA, is equal to 95.24. Next, y_train and y_test are concatenated to the *df_pca_train* and *df_pca_test* data-frames. Finally, the two data-frames are merged to form the *df_pca* data-frame, obtained from the complete preprocessing of the entire dataset.

At this point, the phase of creating the LSTM model for the binary classification between benign and malicious packages is executed. The *df_pca* dataframe, obtained from the preprocessing phase, is converted into two Numpy arrays:

- X: containing, for each row of the data-frame (except the last 50), a set of 50 packets, of which 49 successive packets and the packet in question;
- y: containing all the labels of the data-frame, except for the first 50 rows.

The reasoning behind this choice is explained below: for each flow of 50 packets in array X, the label of the 51st packet is inserted into y, since y represents the array of expected values. Next, these two arrays are split into three parts: training set (70%), validation set (15%), and test set (15%). For each of these sets, it is checked that there are both types of packets (benign and malicious) and that they are present in approximately the same number.

Subsequently, the construction of the sequential model is carried out, with a total of 14 total layers. LSTM layers are added (with an output dimensionality of 80), alternating with Dropout layers (a regulation technique used that randomly sets the inputs to 0, with a frequency equal to 0.3 for each step during training, to prevent overfitting). As the second-last layer, a Dense layer is used, which is deeply connected to the previous layer and which allows changing the dimension of the output vector to 1. The last layer is Activation, sigmoid type.

## 6. Results

The model is trained, with the aid of the training set, in five epochs, with a batch size of 512. Furthermore, the model is also validated. At the latest epoch, the loss and accuracy statistics are as follows:

- Loss for training set: 0.0158.
- Loss for validation set: 0.0167.
- Accuracy for training set: 0.9970.
- Accuracy for validation set: 0.9968.

**Figure 8:**
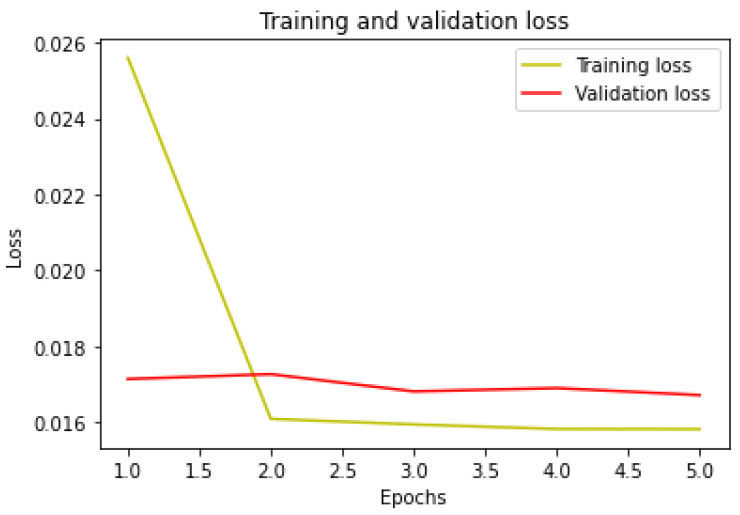
Loss for training and validation set.

In Figure 8, the plot of the loss for each epoch is shown, relative to the training set and the validation set.

**Figure 9:**
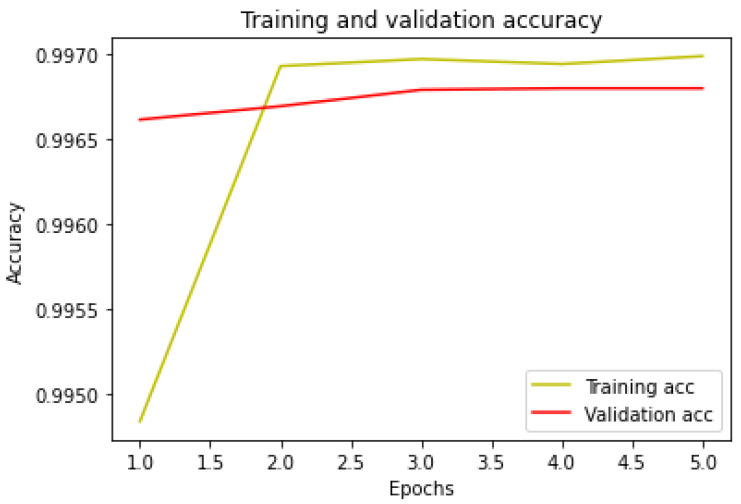
Accuracy for training and validation set.

In Figure 9, the accuracy plot for each epoch is shown, relative to the training set and the validation set.

The curves appear to have an optimal trend, improving for each epoch run.

**Figure 10:**
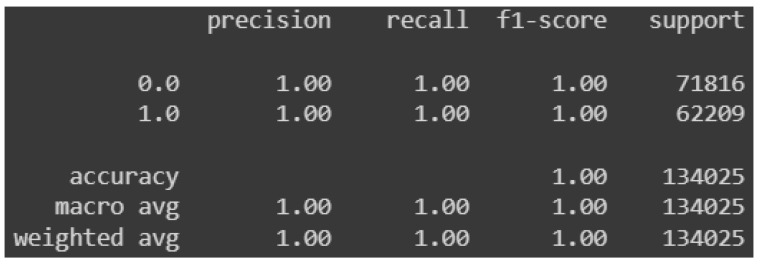
Precision, recall, and F1-score for predictions on test set.

Subsequently, the test set is used to make predictions and compute the loss and accuracy statistics of the model. The accuracy is high for the model proposed in this paper, and approximately equal to 99.71%, while the loss assumes a low value, approximately equal to 0.01. The classification report shown in Figure 10 displays the precision, recall, and F1-score metrics for both packet classes.

**Figure 11:**
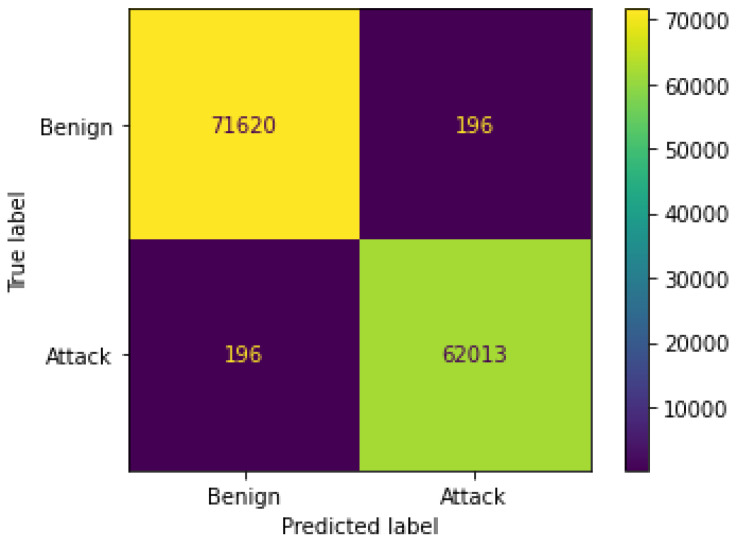
Confusion matrix for predictions on test set.

The metrics take on optimal values. Figure 11, on the other hand, shows the confusion matrix based on the predictions made on the test set:

The statistics represent the number of true positives (TP), which is equal to 62,013; the number of true negatives (TN), which is equal to 71,620; the number of false positives (FP), which is equal to 196; and the number of false negatives (FN), which is equal to 196. The number of FP and FN is very low. Therefore, the model appears to have optimal performance to perform the task for which it was created.

At this point, the model is used within the PN described in the previous section. From the *df_pca* preprocessed dataframe, the last 15% of the data are considered and used to run the entire IDS. During execution, 10 streams of 50 packets are processed at a time. At the end of the execution, 306 packets are blocked, while 204 packets are unblocked. The statistics are as follows:

- Percentage of blocked attack packets: 91.5%.
- Percentage of blocked benign packets: 8.5%.
- Percentage of unblocked attack packets: 20.1%.
- Percentage of unblocked benign packets: 79.9%.

The system works optimally, allowing the blocking of entire streams of packets targeted as malicious.

Table 6 shows a comparison of different literature works performance with respect to the approach described in this paper.

## 7. Conclusions

In this paper, a Long Short-Term Memory (LSTM)-based Intrusion Detection System (IDS) is proposed in which the dataset consists of a labeled group of sequential packets. The instances are time correlated and include a selected set of features representing cumulative data, such as the packet sizes in forward and backward directions and packet flow statistics. Each instance in the dataset is classified as either benign or attack in a binary fashion.

An LSTM network with appropriate hyper-parameters is trained to classify a packet sequence as benign or malicious and recognize attacks in real time. In addition, an Intrusion Prevention System (IPS) is modeled by using a Petri Net (PN) to prevent malicious traffic from flowing through an enterprise network. Simulation results using a publicly available dataset demonstrate that the proposed approach correctly identifies 91.5% of packets predicted as attacks and subsequently discarded by the PN-based IPS.

Future work will focus on improving the proposed model by exploring the use of different ML techniques to further reduce the number of false positives. Also, we plan to focus on time performance to evaluate real-time capabilities of our system in comparison to other IDS methods, and we aim to perform cross-validation to provide a more comprehensive error estimation and better insight into the model’s generalizability.

## Figures and Tables

**Table 1:** State of the art analysis based on the use of IDS 2018 dataset.

Paper	Contribution	Shortcoming
Lin et al. [40]	Dynamic network anomaly detection system using LSTM and Attention Mechanisms for traffic classification.	Class imbalance, training complexity, real-time detection capability, feature selection.
Kim et al. [41]	Development of a CNN-based IDS and comparison with prebuilt RNN techniques.	Dataset dependence, preprocessing overhead, model complexity, performance on imbalanced data, feature selection.
Kanimozhi et al. [42]	Artificial Intelligence-based approach to detect botnet attacks.	Hyper-parameter optimization complexity, scalability issues, dataset limitations, real-time detection capability.
Karatas et al. [43]	Comparison of six ML-based IDSs by using K Nearest Neighbor, Random Forest, Gradient Boosting, Adaboost, Decision Tree, and Linear Discriminant Analysis algorithms; application of Synthetic Minority Oversampling Technique to balance the dataset.	Data imbalance handling, feature selection, dataset limitations, evaluation metrics, generalization.
Kim et al. [44]	Deep learning-based IDS focused on Denial of Service attacks. Usage of Convolutional Neural Networks and Recurrent Neural Networks.	Dataset limitation, class imbalance, generalization, comparison with limited models, hyper-parameter optimization, real-time implementation.
Dey [45]	Deep learning technique based on CNN and LSTM.	Dataset dependency, imbalanced data handling, generalization, adaptability.
Ayachi et al. [46]	Usage of adapted hyper-parameters in an artificial neural network to improve the general accuracy.	Preprocessing overhead, model complexity, generalization.
Gopalan et al. [47]	Analysis of balancing approaches using ML and definition of a taxonomy of the supervised ML techniques applied to train models for classification.	Dataset dependence, preprocessing overhead, imbalanced data handling, generalization, adaptability.

**Table 2:** Dataset structure before PCA.

	0	1	2	3	4
Dst Port	−0.962971	−0.962060	−0.962971	−0.960196	−0.962971
Protocol	−193,135,070	0.003857	−193,135,070	354,091,889	−193,135,070
Flow Duration	15,824,719	−0.113343	15,824,721	−0.422853	15,824,708
Fwd Pkt Len Min	−0.001147	−0.001147	−0.001147	871,499,283	−0.001147
Flow Byts/s	−0.097724	0.055520	−0.097724	79,437,217	−0.097724
Flow Pkts/s	−0.130869	−0.128415	−0.130869	0.432200	−0.130869
Bwd IAT Min	−0.447516	−0.436038	−0.447516	−0.447516	−0.447516
Fwd PSH Flags	−0.002295	−0.002295	−0.002295	−0.002295	−0.002295
FIN Flag Cnt	−0.034175	−0.034175	−0.034175	−0.034175	−0.034175
RST Flag Cnt	0.0	0.0	0.0	0.0	0.0
PSH Flag Cnt	−0.137693	7,262,515	−0.137693	−0.137693	−0.137693
URG Flag Cnt	−0.174805	−0.174805	−0.174805	−0.174805	−0.174805
ECE Flag Cnt	0.0	0.0	0.0	0.0	0.0
Down/Up Ratio	−0.267129	−0.267129	−0.267129	3,319,988	−0.267129
Fwd Seg Size Min	−55,238,334	0.046221	−55,238,334	−41,417,195	−55,238,334
Active Mean	−0.011527	−0.011527	−0.011527	−0.011527	−0.011527
Active Std	−0.002351	−0.002351	−0.002351	−0.002351	−0.002351
Idle Std	0.004111	−0.002575	−0.001406	−0.002575	0.001306

**Table 3:** Dataset structure after PCA.

	0	1	2	3	4
0	5,074,604	0.173635	5,071,693	2,598,003	5,073,123
1	0.162982	4,461,345	0.163206	120,839,396	0.163096
2	37,948,500	−1,053,695	37,948,894	−69,087,613	37,948,694
3	−83,421,649	0.692999	−83,421,672	171,341,563	−83,421,662
4	−116,180,270	−0.736592	−116,180,261	830,636,674	−116,180,265
5	−22,494,404	−1,279,925	−22,494,358	123,686,500	−22,494,383
6	6,406,088	−0.020321	6,406,177	7,649,916	6,406,132
7	6,521,164	−0.516545	6,523,266	14,324,288	6,522,233
8	−79,378,003	−0.632227	−79,378,146	−217,569,203	−79,378,071
9	−1,263,564	0.455594	−1,263,568	−12,495,999	−1,263,565
10	104,454,578	−0.646437	104,453,856	292,079,718	104,454,214
11	21,091,575	−0.109694	21,093,984	60,465,977	21,092,801
12	0.653150	−3,806,546	0.652938	−22,163,114	0.653048
Label	0	0	0	0	0

**Table 4:** LSTM parameters.

Parameter	Value
Input layer size	50
Output layers size	1 (binary)
Activation function	Sigmoid
Number of Hidden Layers	6
Hidden layers size	80
Number of Dropout Layers	6
Drop probability	0.3

**Table 5:** Extracted feature and their description.

Feature	Description
dst_port	Destination port of connection
protocol	Protocol used during connection
fl_dur	Flow duration
tot_l_bw_pkt	Total size of packet in backward direction
fw_pkt_l_min	Minimum size of packet in forward direction
fl_byt_s	flow byte rate, which is number of packets transferred per second
fl_pkt_s	flow packets rate, which is number of packets transferred per second
bw_iat_min	Minimum time between two packets sent in the backward direction
fw_psh_flag	Number of times the PSH flag was set in packets traveling in the forward direction (0 for UDP)
bw_pkt_s	Number of backward packets per second
fin_cnt	Number of packets with FIN
rst_cnt	Number of packets with RST
pst_cnt	Number of packets with PUSH
urg_cnt	Number of packets with URG
down_up_ratio	Download and upload ratio
fw_seg_min	Minimum segment size observed in the forward direction
atv_avg	Mean time a flow was active before becoming idle
atv_std	Standard deviation time a flow was active before becoming idle
idl_std	Standard deviation time a flow was idle before becoming active

**Table 6:** Comparison between current literature and the proposed approach.

Paper	Best Achieved Accuracy	Model
Lin et al. [40]	96.2%	LSTM and Attention Mechanism
Kim et al. [41]	75%	CNN
Kanimozhi et al. [42]	99.97%	Artificial Neural Network
Karatas et al. [43]	99.69%	Adaboost algorithm
	99.66%	Decision Tree
	99.21%	Random Forest
	98.52%	K-Nearest Neighbor
	99.11%	Gradient Boosting
	90.80%	Linear Discriminant Analysis
Kim et al. [44]	91.5%	CNN
Dey [45]	99.98%	Attention-based LSTM
Ayachi et al. [46]	99.91%	Artificial Neural Networks with adapted hyper-parameters
Proposed approach	99.71%	LSTM and Petri Net

## Data Availability

Data are contained within the article.
